# The Association of Rose Bengal with Macrophage Polarization and Oxidative Stress Response in Full-Thickness Excisional and Grafted Burn Wounds: A Porcine In Vivo Study

**DOI:** 10.3390/medicina62040629

**Published:** 2026-03-26

**Authors:** Julia Kleinhapl, Juquan Song, Ye Wang, Kan Nakamoto, Gabor Toro, Isabelle Bergman, Ludwik K. Branski, Steven E. Wolf, Amina El Ayadi

**Affiliations:** 1 Department of Surgery, University of Texas Medical Branch, Galveston, TX 77555, USA; jusong@utmb.edu (J.S.); yewang@utmb.edu (Y.W.); gatoro@utmb.edu (G.T.); lubransk@utmb.edu (L.K.B.); swolf@utmb.edu (S.E.W.); 2 Division of Plastic, Aesthetic and Reconstructive Surgery, Department of Surgery, Medical University of Graz, 8036 Graz, Austria; 3 Department of Anesthesiology, University of Texas Medical Branch, Galveston, TX 77555, USA; kan.nakamoto@gmail.com; 4 John Sealy School of Medicine, University of Texas Medical Branch, Galveston, TX 77555, USA; ibbergma@utmb.edu; 5 Shriners Children’s Texas, Galveston, TX 77550, USA

**Keywords:** burn, wound healing, grafting, Rose Bengal, hydrogel, macrophage polarization, oxidative stress, 4-HNE, CD206

## Abstract

*Background and Objectives*: Burn wounds are associated with delayed healing, infection, and pathological scarring. Effective repair requires tightly regulated immune and oxidative stress responses, including macrophage polarization. This study evaluated the association of the photosensitizer Rose Bengal, delivered in a hydrogel vehicle, with macrophage polarization and oxidative stress after burn injury. *Materials and Methods*: Three female red Duroc pigs underwent full-thickness contact burns followed by excision and autografting. Wounds received 20% Pluronic F-127 hydrogel containing 0.1% Rose Bengal sodium, hydrogel alone, or PBS (phosphate-buffered saline) on days 1, 7, and 14 post-burn. Biopsies from days 7 and 120 were analyzed by immunohistochemistry for pan-macrophage marker, CD206 (M2 macrophages), CD3E (T-cell infiltration), and 4-hydroxynonenal (4-HNE; oxidative stress marker). Mean fluorescence intensity was analyzed using two-way ANOVA with Tukey’s post hoc test (mean ± SD, *p* < 0.05). *Results*: At day 120, Rose Bengal treatment showed higher pan-macrophage expression (0.80 ± 0.07) compared with PBS (0.62 ± 0.10; *p* = 0.0034), whereas the difference versus hydrogel (0.68 ± 0.07; *p* = 0.0628) was not significant. CD206 expression was similarly higher in Rose Bengal-treated wounds (0.77 ± 0.06) compared with PBS (0.62 ± 0.05; *p* = 0.0277); hydrogel also differed from PBS (*p* = 0.0287), without a difference between hydrogel and Rose Bengal. For CD3E, a significant main effect of treatment was observed (F(2,12) = 8.346, *p* = 0.0054), with lower values in Rose Bengal versus PBS at day 120 (*p* = 0.0360). No differences in 4-HNE were detected. *Conclusions*: Rose Bengal–hydrogel treatment was associated with increased macrophage presence and enhanced M2 polarization without increased T-cell infiltration. Effects were significant versus PBS but not hydrogel, suggesting Rose Bengal may contribute to a pro-regenerative immune microenvironment without excessive adaptive activation.

## 1. Introduction

With approximately 30,000 hospital admissions annually in the United States attributable to burn injuries, the management of burn wounds remains a substantial clinical challenge [1]. Burn wounds represent a distinct subtype of complex wounds, characterized by systemic inflammation, local perfusion disturbances, and extensive tissue destruction that impair intrinsic regenerative capacity [2,3]. Despite advances in wound coverage strategies, severe burn injuries often necessitate surgical excision and autologous skin grafting, yet remain associated with delayed healing and complications such as infections [4,5,6]. Effective wound healing depends on a tightly regulated interplay between immune cells, structural proteins, and local tissue factors [7]. Disruption of this balance may result in chronic inflammation, impaired regeneration, and ultimately disfiguring and dysfunctional scarring [7,8,9].

Accordingly, the development of innovative biomaterials for wound coverage aims to reduce healing time, minimize hypertrophic and atrophic scar formation, and improve functional outcomes following wound closure [10]. Such materials are designed to interact with the cellular components involved in the four classical stages of wound healing, while providing structural support for neodermal regeneration [10,11].

Following cutaneous injury, the innate immune system is the first to detect disruption of tissue homeostasis and subsequently shapes the adaptive immune response [12]. The inflammatory phase transitions into proliferative and remodeling stages through tightly coordinated cellular signaling pathways [13]. Among these, macrophage polarization plays a pivotal role [14]. Dysregulated polarization, particularly persistent pro-inflammatory (M1) activation or impaired transition toward reparative (M2) phenotypes, has been associated with delayed healing and pathological scarring [15,16]. Recent studies have emphasized the temporal dynamics of macrophage phenotypic shifts as critical determinants of wound resolution [14].

Oxidative stress represents another key regulator of this delicate balance. Physiological levels of reactive oxygen species (ROS) contribute to antimicrobial defense and act as signaling mediators that promote angiogenesis and cellular proliferation, whereas excessive ROS accumulation leads to tissue damage and impaired regeneration [17]. Therapeutic modulation of oxidative balance therefore represents a potential strategy to enhance wound healing [18,19].

Rose Bengal, a water-soluble xanthene dye that was originally synthesized in the late 19th century, has evolved into a versatile compound with applications across multiple medical disciplines [20,21,22,23]. Its function as a photosensitizer underlies its use in photodynamic therapy (PDT), where activation by green light (approximately 532 nm) generates singlet oxygen and other ROS, leading to cytotoxic and antimicrobial effects [24]. In addition to PDT, Rose Bengal enables photochemical tissue bonding (PTB), in which light-induced ROS promote covalent cross-linking of structural proteins such as collagen type I, thereby enhancing mechanical stability and tissue approximation [25].

Within surgical research, Rose Bengal has demonstrated promising results in promoting tissue repair and wound healing [26,27]. However, its immunomodulatory and oxidative stress-related effects in burn wound healing, particularly in large animal models, remain insufficiently characterized. Therefore, this study aimed to evaluate the association of repeated topical Rose Bengal application on macrophage polarization and oxidative stress response following severe burn injury in a porcine wound healing model.

## 2. Materials and Methods

### 2.1. Study Design and Ethical Approval

The present analysis focuses on the immunohistochemical characterization of specific cellular responses relevant to tissue repair. The work was conducted at the University of Texas Medical Branch (UTMB), Galveston, Texas. The protocol was approved by the Institutional Animal Care and Use Committee (IACUC; #2112070). The experimental period spanned January 2024 to August 2025 and followed institutional and national guidelines for the care and use of laboratory animals. Reporting adheres to the ARRIVE 2.0 guidelines [28].

Three female Red Duroc pigs were purchased from MD Anderson Research Center (Houston, TX, USA) and served as the study population (mean weight at arrival: 36.7 kg). Animals were housed in stainless steel caging measuring 95″ × 75.5″ with a rubber ergonomic mat provided for resting. Ambient temperature and humidity were maintained at 61–81 °F (target: 73 °F) and 30–70%, respectively. Housing density included two animals per cage during study periods in which two cycles of pigs overlapped and until the animals reached a weight of 100 kg. During study phases in which only one pig was enrolled at a time, or once animals exceeded this weight, pigs were housed individually in adjacent cages allowing visual, auditory, and limited physical contact.

Environmental enrichment included plastic and rubber toys that were rotated every two weeks. In addition, pigs received fruits and vegetables once daily on weekdays. When housed individually, enrichment also included 10–15 min of human interaction twice daily. Animals were fed once daily in the morning with a standard laboratory porcine grower diet (LabDiet #5084). Drinking water (city water) was available ad libitum via the cage watering system. Cages were cleaned twice daily. A one-week acclimatization period preceded all experimental procedures.

### 2.2. Burn Injury, Excision and Grafting Model

On day 0, sixteen full-thickness contact burns (2.5 × 2.5 cm) were induced on the paravertebral dorsum using a custom-built aluminum burn device heated to 200 °C (±5 °C). Total body surface area (TBSA) was calculated using a weight-based formula (TBSA (m^2^) = 0.121 × weight (kg)^0.575^) originally described by Wachtel et al., with final burn sizes expressed as a percentage of the TBSA [29,30]. The study followed an established full-thickness contact burn model with subsequent excision and grafting as established at our institution [31]. On day 1 (24 h after burn), full-thickness tangential excision was performed, extending 1 cm beyond the burn area to achieve a 3.5 × 3.5 cm square. Autologous split-thickness skin grafts (thickness 30/1000 inch) were harvested from the dorsal thighs using an air dermatome, meshed 1:4, and secured to the wound bed using a stapling technique. Wounds were cleansed prior to excision with povidone-iodine and saline under a sterile technique. All procedures were performed under anesthesia with pre-analgesia. Treatments (4 mL per wound) were applied directly to the wound bed on top of the graft using a syringe according to randomization and secured using a bolster dressing technique to ensure uniform pressure and promote graft take [32].

On day 7, bolster dressings were removed and wounds were cleaned with sterile saline. Treatments were re-applied on day 7 and 14. Dressing changes, wound assessments, and punch biopsies (0.6 cm diameter) were performed on days 7, 14, 28, 60, 90, and 120, with a permitted variability of ±2 days for logistical reasons. Biopsies were collected from each wound and from adjacent unburned skin as control tissue. For the present analysis, biopsies from day 7 and day 120 were evaluated, representing early and late phases of wound healing, respectively.

On day 120, animals were euthanized under deep anesthesia by intravenous administration of saturated potassium chloride (KCL) solution (MilliporeSigma, Burlington, MA, USA) until cardiac arrest was achieved, lfollowed by bilateral thoracotomy in accordance with institutional animal protocol and the American Veterinary Medical Association (AVMA) guidelines [33].

One animal was euthanized on day 28 due to non-treatment-related clinical decline. Necropsy revealed no treatment-associated findings and identified a rectal stricture that appeared to have caused intestinal obstruction. Thorough macroscopic examination of all internal organs did not reveal any abnormalities. The predefined sampling strategy (*n* = 5 wounds per antibody) was maintained using the remaining animals.

### 2.3. Treatments

Three treatments were applied: (1) 20% Pluronic F-127 vehicle hydrogel (Sigma-Aldrich, St. Louis, MO, USA), (2) 0.1% Rose Bengal sodium-loaded 20% Pluronic F-127 vehicle hydrogel, or (3) phosphate-buffered saline (PBS). Hydrogels were produced under sterile conditions in our laboratory. The Rose Bengal sodium solution was provided by Provectus Biopharmaceuticals, Inc. (Knoxville, TN, USA), and PBS, which served as the control treatment, was obtained commercially. Hydrogels were prepared 24 h prior to application and maintained at 4 °C to ensure homogenous solubility of the drug in the hydrogel. The mixture is moved to 37 °C one hour before application to ensure appropriate viscosity. Rose Bengal-loaded hydrogel was protected from ambient light to prevent premature photoactivation. In this study, green-light activation was not performed. Instead, ambient light exposure combined with repetitive application was used to evaluate whether this approach could serve as an alternative activation strategy. Treatments were randomized across a total of 46 wounds (Rose Bengal-loaded hydrogel *n* = 18, hydrogel *n* = 14, PBS *n* = 14) and applied on days 1, 7, and 14. The experimental unit was defined as the individual wound. Wounds were distributed across three animals at the early timepoint and two animals at the late timepoint. Experimental units (wounds) were randomly allocated to treatment groups within each animal using computer-generated randomization in Microsoft Excel (Microsoft Corp., Redmond, WA, USA). Random numbers were generated using the RAND() function, and wounds were assigned based on the ascending order of the generated values. No formal a priori sample size calculation was performed. Sample size was determined based on feasibility and experience with prior porcine full-thickness burn wound studies using comparable experimental designs at our institution.

### 2.4. Anesthesia, Analgesia and Animal Monitoring

Long-acting buprenorphine (0.2–0.6 mg/kg) was administered subcutaneously prior to each surgical session for analgesia. Animals received pre-anesthetic sedation with a TKX cocktail (prepared by reconstituting one vial of Telazol with 5 mL ketamine [100 mg/mL] and 5 mL xylazine [100 mg/mL]) administered intramuscularly at a dose of 0.025 mL/kg. Following adequate sedation, animals were endotracheally intubated, and anesthesia was induced and maintained with inhalational isoflurane (0.1–5%). Procedures lasted approximately 2–3 h per session. Physiological parameters, including heart rate, body temperature, and pulse oximetry, were continuously monitored by trained anesthesiology personnel. Given the small extent of TBSA (~1%), no burn-related fluid resuscitation was required during or after the burn procedure. However, animals received intravenous maintenance fluids (0.9% saline) intraoperatively during excision and grafting procedures.

Postoperative monitoring was performed and documented twice a day by research personnel. This included assessments for signs of pain (behavioral changes such as altered posture, lack of tail movement, distressed movement or vocalization, decreased food intake, low urine and fecal output, and reduced activity), bleeding, wound infection (macroscopic changes such as erythema, edema, or increased local skin temperature), and systemic illness (changes in activity level or food intake). Immediately after surgery, animals were observed in their cages until fully recovered from anesthesia, indicated by the ability to stand and resume food intake. Vivarium staff additionally monitored the animals twice daily, and any health concerns were promptly reported to the veterinary team. No additional interventions or medications known to affect wound healing were administered during the study. Predefined humane endpoints included uncontrollable postoperative bleeding; respiratory compromise (e.g., distress or hypoventilation); persistent pain or distress unresponsive to analgesic treatment; and reduced activity with failure to rise and/or weight loss exceeding 20% of initial body weight despite supportive measures (e.g., additional feeding, analgesia, and stimulation) for more than 48 h.

### 2.5. Sample Processing and Immunohistochemistry (IHC)

Biopsies were obtained under sterile conditions, embedded in optimal cutting temperature (OCT) compound and snap-frozen in liquid nitrogen immediately after collection. For immunohistochemistry, 10 µm cryosections were fixed in 4% paraformaldehyde for 30 min, incubated in antigen retrieval solution (1:10 in nanopure water) for 45 min at 70 °C, and permeabilized with 0.1% Triton X-100 in PBS for 20 min. After three PBS washes, sections were blocked with 5% donkey serum for one hour to reduce nonspecific binding. Tissue sections were then incubated with primary antibodies overnight at 4 °C (Table 1), followed by species-appropriate secondary antibodies the next day (Alexa Fluor 488 goat anti-mouse (cat# A11001, Invitrogen, Thermo Fisher Scientific, Waltham, MA, USA) for macrophage and 4-HNE antibodies; Alexa Fluor 488 donkey anti-rabbit (cat# A21206, Invitrogen, Thermo Fisher Scientific, Waltham, MA, USA) for CD206 and CD3E antibodies). Nuclei were counterstained with 4′,6-diamidino-2-phenylindole (DAPI 1:1000), and sections were mounted using a xylene-based mounting medium.

Four primary antibodies targeting macrophages (pan-macrophage marker), CD206 (mannose receptor), CD3E (T-cell receptor complex subunit epsilon), and 4-hydroxynonenal (4-HNE) were analyzed at early (day 7 post-burn) and late (day 120 post-burn) timepoints to compare acute versus subacute-to-chronic cellular responses (Table 1).

Images were acquired using an Olympus BX53-F microscope (Olympus, Tokyo, Japan) equipped with a U-RFL-T mercury lamp power supply at 40× magnification. Exposure settings were kept constant across all samples, with antibody signals captured at 20–30 ms and DAPI at 250–500 µs. Mean fluorescence intensity was quantified using Fiji (ImageJ version 1.54p; National Institutes of Health, Bethesda, MD, USA) with a batch-analysis macro modified to apply a fixed threshold range (lower limit 20, upper limit 168) and exclude the scale bar from the analysis [34]. The same threshold settings were applied across all images. For each antibody, *n* = 5 biopsies of wounds per treatment group were stained and analyzed, with three regions of interest (ROIs) randomly selected within the papillary dermis immediately beneath the epidermis. ROI values were normalized to DAPI and averaged to obtain a normalized fluorescence ratio. For each antibody, control biopsies of unburned, untreated skin were stained and imaged (*n* = 4 per group) to establish a baseline. Negative control staining was performed by omitting the primary antibody and incubating sections with the respective secondary antibody under identical experimental conditions. No specific staining was observed in negative control sections.

Due to the visibly distinct characteristics of the treatments, investigators involved in the surgical procedures and treatment application (up to four researchers) were aware of group allocation at the time of allocation and during conduct of the experiments. Image acquisition and quantitative analysis were performed without reference to treatment allocation to minimize assessment bias.

### 2.6. Statistical Analysis

Group comparisons were performed using repeated measures two-way ANOVA followed by Tukey’s post hoc test for multiple comparisons in GraphPad Prism (version 10.0.0; GraphPad Software, San Diego, CA, USA). Data are presented as mean ± SD. A *p*-value < 0.05 was considered statistically significant.

## 3. Results

The mean TBSA at the time of burn was 0.96 ± 0.05 m^2^, with the standardized burn size of 100 cm^2^ corresponding to 1.04 ± 0.06% TBSA.

### 3.1. Pan-Macrophage Marker Expression

**Figure 1:**
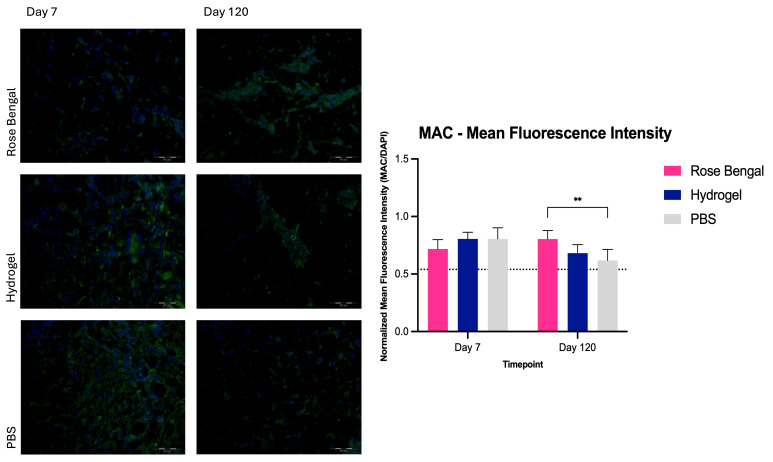
Pan-macrophage marker expression in Rose Bengal-, hydrogel-, and PBS-treated burn wounds at day 7 and day 120 post-injury. Mean fluorescence intensity of the pan-macrophage marker was quantified and expressed as a normalized fluorescence ratio relative to DAPI. The dotted line indicates the mean value of control (uninjured) skin samples. Data are presented as mean ± SD. **, *p* < 0.01.

Two-way repeated measures ANOVA revealed a significant interaction between treatment and time (F(2,12) = 7.679, *p* = 0.0071). A significant main effect of time was observed (F(1,12) = 6.354, *p* = 0.0269), whereas no significant main effect of treatment was detected (F(2,12) = 1.004, *p* = 0.3954). At day 7 post-burn, mean pan-macrophage marker expression was 0.72 ± 0.08 in Rose Bengal-treated wounds, 0.81 ± 0.06 in hydrogel-treated wounds, and 0.81 ± 0.10 in PBS-treated wounds. Post hoc analysis revealed no significant differences between treatment groups at this timepoint. At day 120 post-burn, Rose Bengal treatment demonstrated a significantly higher mean normalized fluorescence ratio for pan-macrophage expression (0.80 ± 0.07) compared with PBS (0.62 ± 0.10; *p* = 0.0034), whereas the difference compared with hydrogel (0.68 ± 0.07; *p* = 0.0628) did not reach statistical significance. No significant difference was observed between hydrogel and PBS (*p* = 0.4238) (Figure 1).

### 3.2. CD206 Receptor Expression

**Figure 2:**
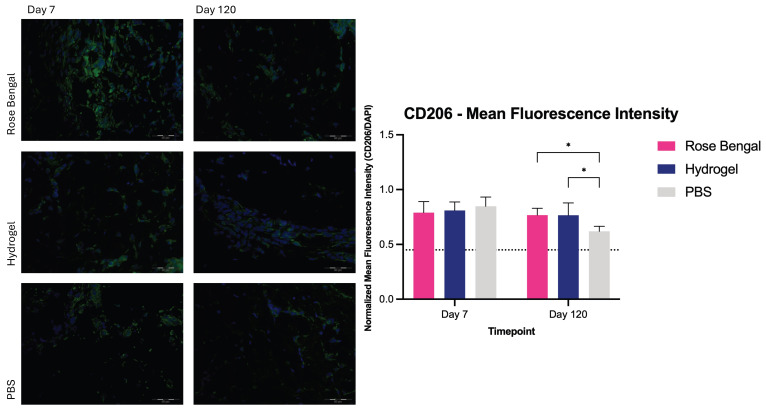
CD206 expression in Rose Bengal-, hydrogel-, and PBS-treated burn wounds at day 7 and day 120 post-injury. Mean fluorescence intensity of CD206 staining was quantified and expressed as a normalized fluorescence ratio relative to DAPI. The dotted line indicates the mean value of control (uninjured) skin samples. Data are presented as mean ± SD. *, *p* < 0.05.

Two-way repeated-measures ANOVA revealed a significant interaction between treatment and time (F(2,12) = 4.407, *p* = 0.0367). A significant main effect of time was observed (F(1,12) = 10.06, *p* = 0.0080), whereas no significant main effect of treatment was detected (F(2,12) = 1.216, *p* = 0.3305). At day 7 post-burn, the mean normalized fluorescence ratio was 0.79 ± 0.10 in Rose Bengal-treated wounds, 0.81 ± 0.08 in hydrogel-treated wounds, and 0.85 ± 0.08 in PBS-treated wounds. Post hoc analysis revealed no significant differences between treatment groups at this timepoint. At day 120 post-burn, Rose Bengal-treated wounds demonstrated significantly higher CD206 expression (0.77 ± 0.06) compared with PBS (0.62 ± 0.05; *p* = 0.0277). Hydrogel-treated wounds (0.77 ± 0.11) also showed significantly higher expression compared with PBS (*p* = 0.0287); however, hydrogel treatment did not significantly differ from Rose Bengal treatment (Figure 2).

### 3.3. CD3E-Positive T-Cell Infiltration

**Figure 3:**
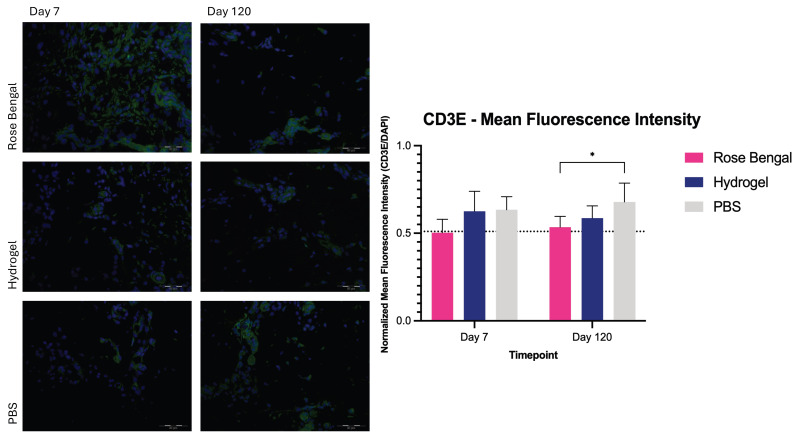
CD3E-positive T-cell infiltration in Rose Bengal-, hydrogel-, and PBS-treated burn wounds at day 7 and day 120 post-injury. Mean fluorescence intensity of CD3E staining was quantified and expressed as a normalized fluorescence ratio relative to DAPI. The dotted line indicates the mean value of control (uninjured) skin samples. Data are presented as mean ± SD. *, *p* < 0.05.

Two-way repeated-measures ANOVA revealed no significant interaction between treatment and time (F(2,12) = 0.55, *p* = 0.5862). No significant main effect of time was observed (F(1,12) = 0.13, *p* = 0.7279). However, a significant main effect of treatment was detected (F(2,12) = 8.346, *p* = 0.0054). At day 7 post-burn, the mean normalized fluorescence ratio for CD3E-positive T cells was 0.63 ± 0.11 in the hydrogel group, 0.50 ± 0.08 in the Rose Bengal group, and 0.63 ± 0.08 in the PBS group. At day 120 post-burn, mean values were 0.59 ± 0.07 in the hydrogel group, 0.53 ± 0.06 in the Rose Bengal group, and 0.68 ± 0.11 in the PBS group. Although mean CD3E expression tended to be lower in the Rose Bengal group, post hoc analysis demonstrated a significant difference only between Rose Bengal-treated wounds and those treated with PBS at day 120 (*p* = 0.0360) (Figure 3).

### 3.4. 4-HNE Expression

**Figure 4:**
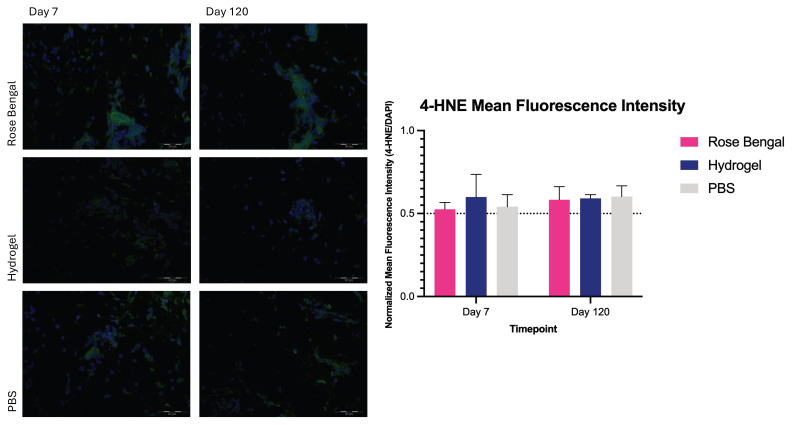
4-HNE expression in Rose Bengal-, hydrogel-, and PBS-treated burn wounds at day 7 and day 120 post-injury. Mean fluorescence intensity of 4-HNE staining was quantified and expressed as a normalized fluorescence ratio relative to DAPI. The dotted line indicates the mean value of control (uninjured) skin samples. Data are presented as mean ± SD.

Two-way repeated-measures ANOVA revealed no significant interaction between treatment and time (F(2,24) = 0.62, *p* = 0.5489). No significant main effect of time was observed (F(1,24) = 1.64, *p* = 0.2122), nor was there a significant main effect of treatment (F(2,24) = 0.69, *p* = 0.5123). At day 7 post-burn, the mean normalized fluorescence ratio was 0.53 ± 0.04 in the Rose Bengal group, 0.60 ± 0.14 in the hydrogel group, and 0.54 ± 0.07 in the PBS group. At day 120 post-burn, mean values were 0.58 ± 0.08 in the Rose Bengal group, 0.59 ± 0.02 in the hydrogel group, and 0.60 ± 0.07 in the PBS group. No significant differences were observed between treatment groups at either timepoint (Figure 4).

## 4. Discussion

This is the first porcine wound healing study to investigate the association of a multidose application of Rose Bengal with macrophage polarization, immune cell infiltration, and oxidative stress after burn. Whereas previous studies primarily relied on green light (540–560 nm) activation of a single dose Rose Bengal application, the present study used ambient light conditions with three repetitive applications [22,25,35]. As a non-light-activated intralesional treatment approach, a 10% solution of Rose Bengal (PV-10) has demonstrated promising results in promoting tumor regression and disease control in patients with metastatic melanoma [36].

Our results suggest that Rose Bengal treatment without controlled green light activation, but with repetitive application, may still induce photodynamic activity and influence macrophage behavior. Specifically, Rose Bengal treatment was associated with increased long-term M2 macrophage polarization, reflected by the highest CD206 levels observed at day 120 post-burn compared with other treatments, reaching statistical significance versus PBS. In parallel, pan-macrophage marker expression also peaked in Rose Bengal-treated wounds at this later timepoint and followed the same significance pattern, showing a significant difference compared with PBS but not hydrogel. Together, these findings suggest a relative enrichment of M2-polarized macrophages in Rose Bengal-treated wounds during the chronic remodeling phase of burn wound healing. Although a clear superiority over hydrogel alone was not demonstrated, the observed trend toward higher M2 macrophage expression in the Rose Bengal group raises the possibility that its immunomodulatory effects may be further enhanced under green light activation rather than ambient light activation alone.

While both M1 and M2 macrophage phenotypes are essential for successful wound healing, a transition from M1 to M2 following the initial inflammatory phase is necessary for M2 macrophages to take over the secretion of anti-inflammatory mediators and growth factors. In contrast, M1 macrophages primarily exert pro-inflammatory functions and are associated with phagocytosis and the induction of apoptosis in damaged or infected cells. M2 macrophages, on the other hand, are commonly associated with tissue repair and remodeling rather than persistent pro-inflammatory activation [37,38]. Hesketh et al. discussed the importance of timing in the transition from M1 to M2 macrophages, noting that delays or premature shifts may influence the progression from acute to chronic wounds [16]. They further describe how early or excessive M2 dominance may affect scarring, suggesting that a moderate, but not extensive, M2 phenotype presence is beneficial for preventing scar formation, whereas excessive or prolonged M2 activity may promote fibrosis through fibroblast overactivation and TGF-β signaling. Therefore, the observed increase in CD206 expression at the later timepoint may indicate a shift toward a more pro-regenerative immune microenvironment, particularly in combination with the lower CD3E expression levels observed in Rose Bengal-treated wounds. CD3E-positive T-cell expression was close to baseline in Rose Bengal-treated wounds, whereas higher expression levels were observed in hydrogel- and PBS-treated wounds, reaching statistical significance in the latter. This pattern may suggest that PBS treatment alone is associated with more sustained inflammatory activity even 120 days after burn, while hydrogel dressings, particularly those containing Rose Bengal, may contribute to attenuation of prolonged immune activation.

Previous studies have suggested that hydrogel-based materials can promote M2 polarization and accelerate burn wound healing. For example, Zhao et al. showed in a murine model that a multifunctional gelatin scaffold containing magnesium and tannic acid accelerated burn wound healing by shortening healing time [39]. However, translational in vivo data from large-animal models remain limited. The present study provides large-animal evidence using a porcine model, which more closely resembles human skin architecture and wound healing characteristics compared with rodent models [40].

Similar to macrophage polarization, oxidative stress and reactive oxygen species (ROS) play a pivotal role in wound healing [18,38]. A fine balance exists between low-level accumulation of ROS, which may promote wound healing through pathogen inactivation within the wound bed, and high levels, which induce oxidative stress and activate destructive pathways, thereby hindering wound healing [41]. Analogous to macrophage dynamics, timing and dosing are critical. Low levels of ROS have been shown to accelerate healing by initiating inflammation, mediating angiogenesis, and contributing to pathogen depletion [19].

Rose Bengal acts as a type II photosensitizer that, upon green light activation (532 nm), converts triplet oxygen into singlet oxygen and thereby generates ROS [42,43]. This mechanism underlies photodynamic therapy (PDT), which has so far been primarily applied in oncologic and ophthalmologic settings [24,35,44]. In dermatological and wound healing contexts, Wang et al., for example, induced a moderate increase in ROS using a Rose Bengal-containing hydrogel under light activation and reported improved wound healing outcomes with antibacterial effects in a murine wound healing model [45]. Accordingly, we have previously shown in an excisional murine model that Rose Bengal photoactivation with monochromatic green light increases ROS generation at days 3 and 7 after injury before returning to baseline levels by day 14 [46].

Our results demonstrate above-baseline levels of 4-HNE in all treatment groups at both timepoints, indicating sustained oxidative stress after burn in this model, without significant differences among treatments or timepoints. 4-HNE (4-hydroxynonenal) accumulates during phases of oxidative stress as a byproduct of membrane lipid peroxidation and therefore serves as a surrogate marker of oxidative stress [47]. The overall tendency toward increased 4-HNE levels at the later timepoint may indicate that remodeling processes inherent to burn wound healing are still ongoing. At day 7, the lowest expression was observed in the Rose Bengal-treated group, with levels closest to baseline, potentially suggesting that Rose Bengal application in the acute phase may be associated with lower oxidative stress and reduced inflammation compared to other treatments. This again raises the question of whether green light activation would further modulate this response, potentially resulting in higher 4-HNE levels. Given the similar overall pattern across treatment groups, it remains uncertain whether the measured 4-HNE intensities primarily reflect burn-induced oxidative stress rather than treatment-specific effects. If Rose Bengal had induced substantial additional ROS generation under ambient light conditions, differential 4-HNE levels compared with hydrogel and PBS would have been expected. Future studies with larger sample sizes and variations in treatment modalities, including different dosing strategies and light activation conditions, may further clarify the temporal pattern of 4-HNE expression after burn injury and elucidate potential treatment-specific effects on oxidative stress during burn wound healing.

As part of its photosensitizing properties, Rose Bengal has also been described as a mediator of photochemical tissue bonding (PTB), promoting collagen cross-linking upon green light (532 nm) exposure [25]. This light-induced cross-linking may enhance the structural stability of collagen-rich tissues and has been investigated in various preclinical repair models. Ni et al., for example, reported improved Achilles tendon repair following light activation of a Rose Bengal-enriched electrospun nanofiber graft material in a rabbit model [26]. Ding et al. further evaluated a Rose Bengal-light-activated amniotic membrane for the repair of flexor digitorum profundus tendons in Subei chickens, demonstrating improved joint mobility and reduced tissue inflammation compared with conventional Kessler suturing [27].

These structural and photochemical characteristics suggest potential relevance for tissue stabilization in complex wounds. However, data evaluating Rose Bengal-loaded hydrogels in grafted burn wounds, particularly with regard to immune modulation and oxidative stress-associated tissue alterations, remain scarce. Moreover, the effect of Rose Bengal without green-light activation in a wound healing model over an extended timeframe, as investigated here up to 120 days, has not previously been described. The present study therefore expands upon existing knowledge by examining these parameters in a clinically relevant porcine burn model.

In an era in which biomaterials and advanced wound dressings are rapidly evolving, particular importance lies in the careful adaptation of such materials for burn wound management [48]. The translational value of our study lies in improving the understanding of hydrogel-based dressings in the context of burn wound healing, particularly through the modulation of key biological processes such as macrophage polarization and oxidative stress responses. Hydrogels have already demonstrated benefits in promoting wound healing by maintaining a moist wound environment and supporting tissue regeneration [49]. The incorporation of biologically active components into these materials represents a promising strategy for improving outcomes in complex wounds such as burns. In the future, such approaches may contribute to the development of more individualized wound dressings tailored to specific wound environments and stages of healing. Hydrogels, particularly Pluronic-based systems such as the one used in our study, may also represent a relatively simple and potentially cost-effective material compared with more complex engineered biomaterials, which could be advantageous for broader clinical translation. Extended investigations are necessary to evaluate the therapeutic potential of Rose Bengal in wound healing under different treatment conditions, including varying dosing strategies and light activation modalities, in order to determine whether it may serve as an effective bioactive component for burn wound dressing.

As not all burn patients are treated in specialized burn centers, burn care benefits from accessible and cost-effective materials. In fact, Zonies et al. reported that only about one quarter of burn patients are treated at a verified burn center [50]. Hence, specialized care and advanced materials are often limited to large, designated burn facilities. In many settings, dedicated green-light sources for hydrogel activation may not be readily available. This formed the rationale for the present approach, namely, to investigate whether the effects of Rose Bengal could also be achieved through ambient light activation. If feasible, this would support the development of an affordable and easily implementable hydrogel-based wound dressing with an active component.

While this study provides preliminary evidence that Rose Bengal may influence macrophage polarization and cellular pathways under ambient light conditions, further investigation is required to confirm these findings and translate them into clinical outcomes. The present study focused on immunohistochemical responses and demonstrated associations with M2-dominant macrophage presence and reduced immune cell infiltration. Although Rose Bengal-loaded hydrogel demonstrated superior results compared with PBS in association with macrophage polarization and immune cell infiltration, no significant differences were observed compared with hydrogel alone. Hydrogel treatment itself did not significantly differ from PBS in the respective analyses. This raises the question of whether Rose Bengal may require adjustment in concentration, application frequency, or controlled green-light activation to more clearly distinguish its effect from hydrogel treatment alone, which served as the vector control. Nevertheless, the fact that Rose Bengal-loaded hydrogel, but not hydrogel alone, demonstrated significant differences compared with PBS suggests that the addition of Rose Bengal may contribute to the observed immunomodulatory effects.

We acknowledge the limitations of this translational study, including the lack of direct clinical outcome measures and comprehensive cellular phenotyping. Expanded antibody panels, quantitative cell-type and subset analyses, and functional outcome assessments may further refine and validate these findings.

## 5. Conclusions

This is the first study to examine the association between a Rose Bengal-loaded hydrogel, M2 macrophage polarization, and oxidative stress response in an excisional grafted porcine burn model. Rose Bengal treatment was associated with increased pan-macrophage and M2 macrophage presence, particularly in comparison with PBS treatment. In addition, Rose Bengal-treated wounds remained close to baseline with regard to CD3-positive T-cell infiltration at both timepoints. Although differences between Rose Bengal and hydrogel alone were not consistently significant, these findings suggest that Rose Bengal may contribute to macrophage polarization without marked adaptive immune activation, thereby potentially supporting balanced remodeling rather than sustained inflammatory activity.

## Figures and Tables

**Table 1:** Antibodies used for Immunohistochemistry. GAM, goat anti-mouse; DAR, donkey anti-rabbit.

Antibody	Manufacturer (Catalogue Number)	Host Species	Target/Specificity	Dilution	Secondary Antibody
Mouse anti pig macrophages antibody, clone BA4D5 Monoclonal Antibody (IgG)	Bio-Rad Laboratories (Hercules, CA, USA) (MCA2317GA)	Mouse	Pig/porcine cells of the monocyte/macrophage lineage	1:50	GAM 1:250
MMR/CD206/Mannose Receptor Antibody—BSA Free Polyclonal Antibody (IgG)	Novus Biologicals (Centennial, CO, USA) (NBP1-90020)	Rabbit	Human, Mouse, Porcine, Primate/CD206 receptor	1:50	DAR 1:250
CD3E Polyclonal Antibody (IgG)	Bioss Antibodies (Woburn, MA, USA) (BS-10498R)	Rabbit	CD3 protein (T-cell receptor-CD3 complex) Cross-reactive species: mouse Predicted Cross-reactive species: pig	1:100	DAR 1:250
4-Hydroxynonenal Antibody Monoclonal Mouse, Clone # 198960 (IgG2B)	Bio-Techne (Minneapolis, MN, USA) (MAB3249)	Mouse	4-Hydroxynonenal adducts of histidine residues	1:50	GAM 1:250

## Data Availability

The datasets presented in this article are available upon request. Requests to access the datasets should be directed to Amina El Ayadi (amelayad@utmb.edu).

## Abbreviations

The following abbreviations are used in this manuscript:

ROS	Reactive oxygen species
PDT	Photodynamic therapy
PTB	Photochemical tissue bonding
AVMA	American Veterinary Medical Association
PBS	Phosphate-buffered saline
IHC	Immunohistochemistry
OCT	Optimal cutting temperature compound
DAPI	4′,6-diamidino-2-phenylindole
GAM	Goat-anti-mouse
DAR	Donkey-anti-rabbit
ROI	Region of interest
4-HNE	4-Hydroxynenonal

## References

[1] American Burn Association (2026). Burn Incidence & Treatment in the U.S. Fact Sheet.

[2] Markiewicz-Gospodarek A., Kozioł M., Tobiasz M., Baj J., Radzikowska-Büchner E., Przekora A. (2022). Burn Wound Healing: Clinical Complications, Medical Care, Treatment, and Dressing Types: The Current State of Knowledge for Clinical Practice. Int. J. Environ. Res. Public Health.

[3] Burgess M., Valdera F., Varon D., Kankuri E., Nuutila K. (2022). The Immune and Regenerative Response to Burn Injury. Cells.

[4] Radzikowska-Büchner E., Łopuszyńska I., Flieger W., Tobiasz M., Maciejewski R., Flieger J. (2023). An Overview of Recent Developments in the Management of Burn Injuries. Int. J. Mol. Sci..

[5] Wang Y., Beekman J., Hew J., Jackson S., Issler-Fisher A.C., Parungao R., Lajevardi S.S., Li Z., Maitz P.K. (2018). Burn injury: Challenges and advances in burn wound healing, infection, pain and scarring. Adv. Drug Deliv. Rev..

[6] van den Bosch A.S., Verwilligen R.A.F., Pijpe A., Bosma E., van der Vlies C.H., Lucas Y., Burchell G.L., van Zuijlen P.P.M., Middelkoop E. (2024). Outcomes of dermal substitutes in burns and burn scar reconstruction: A systematic review and meta-analysis. Wound Repair Regen..

[7] Peña O.A., Martin P. (2024). Cellular and molecular mechanisms of skin wound healing. Nat. Rev. Mol. Cell Biol..

[8] Zielińska M., Pawłowska A., Orzeł A., Sulej L., Muzyka-Placzyńska K., Baran A., Filipecka-Tyczka D., Pawłowska P., Nowińska A., Bogusławska J. (2023). Wound Microbiota and Its Impact on Wound Healing. Int. J. Mol. Sci..

[9] Finnerty C.C., Jeschke M.G., Branski L.K., Barret J.P., Dziewulski P., Herndon D.N. (2016). Hypertrophic scarring: The greatest unmet challenge after burn injury. Lancet.

[10] Downer M., Berry C.E., Parker J.B., Kameni L., Griffin M. (2023). Current Biomaterials for Wound Healing. Bioengineering.

[11] Song Y.T., Liu P.C., Zhou X.L., Chen Y.M., Wu W., Zhang J.Y., Li L.J., Xie H.Q. (2024). Extracellular matrix-based biomaterials in burn wound repair: A promising therapeutic strategy. Int. J. Biol. Macromol..

[12] Strbo N., Yin N., Stojadinovic O. (2014). Innate and Adaptive Immune Responses in Wound Epithelialization. Adv. Wound Care.

[13] Gurtner G.C., Werner S., Barrandon Y., Longaker M.T. (2008). Wound repair and regeneration. Nature.

[14] Sharifiaghdam M., Shaabani E., Faridi-Majidi R., De Smedt S.C., Braeckmans K., Fraire J.C. (2022). Macrophages as a therapeutic target to promote diabetic wound healing. Mol. Ther..

[15] Li M., Hou Q., Zhong L., Zhao Y., Fu X. (2021). Macrophage Related Chronic Inflammation in Non-Healing Wounds. Front. Immunol..

[16] Hesketh M., Sahin K.B., West Z.E., Murray R.Z. (2017). Macrophage Phenotypes Regulate Scar Formation and Chronic Wound Healing. Int. J. Mol. Sci..

[17] Bryan N., Ahswin H., Smart N., Bayon Y., Wohlert S., Hunt J.A. (2012). Reactive oxygen species (ROS)—A family of fate deciding molecules pivotal in constructive inflammation and wound healing. Eur. Cell Mater..

[18] Wang G., Yang F., Zhou W., Xiao N., Luo M., Tang Z. (2023). The initiation of oxidative stress and therapeutic strategies in wound healing. Biomed. Pharmacother..

[19] Ukaegbu K., Allen E., Svoboda K.K.H. (2025). Reactive Oxygen Species and Antioxidants in Wound Healing: Mechanisms and Therapeutic Potential. Int. Wound J..

[20] Neckers D.C. (1989). Rose Bengal. J. Photochem. Photobiol. Chem..

[21] Ostańska E., Barnaś E., Bartusik-Aebisher D., Dynarowicz K., Szpunar M., Skręt-Magierło J., Aebisher D. (2022). Histopathological Analysis of the Effect of Photodynamic Action on Post-Chemotherapy Excised Breast Cancer Tissue. Medicina.

[22] Lee J.S., Lee B.I., Park C.B. (2015). Photo-induced inhibition of Alzheimer’s β-amyloid aggregation in vitro by rose bengal. Biomaterials.

[23] Aydın E., Dicle Y., Kaçtaş Ş., Gümüş A.F. (2023). Detection of Human Brucellosis by Brucellacapt and Rose Bengal Test in the Endemic Area. New Trends Med. Sci..

[24] Naranjo A., Arboleda A., Martinez J.D., Durkee H., Aguilar M.C., Relhan N., Nikpoor N., Galor A., Dubovy S.R., Leblanc R. (2019). Rose Bengal Photodynamic Antimicrobial Therapy (RB-PDAT) for Patients with Progressive Infectious Keratitis: A Pilot Clinical Study. Am. J. Ophthalmol..

[25] Wertheimer C.M., Elhardt C., Kaminsky S.M., Pham L., Pei Q., Mendes B., Afshar S., Kochevar I.E. (2019). Enhancing Rose Bengal-Photosensitized Protein Crosslinking in the Cornea. Investig. Ophthalmol. Vis. Sci..

[26] Ni T., Senthil-Kumar P., Dubbin K., Aznar-Cervantes S.D., Datta N., Randolph M.A., Cenis J.L., Rutledge G.C., Kochevar I.E., Redmond R.W. (2012). A photoactivated nanofiber graft material for augmented Achilles tendon repair. Lasers Surg. Med..

[27] Ding B., Wang X., Yao M. (2019). Photochemical Tissue Bonding Technique for Improving Healing of Hand Tendon Injury. Surg. Innov..

[28] Percie du Sert N., Hurst V., Ahluwalia A., Alam S., Avey M.T., Baker M., Browne W.J., Clark A., Cuthill I.C., Dirnagl U. (2020). The ARRIVE guidelines 2.0: Updated guidelines for reporting animal research. PLoS Biol..

[29] Wachtel T.L., McCahan G.R., Watson W.I., Gorman M. (1972). Determining the Surface Areas of Miniature Swine and Domestic Swine by Geometric Design—A Comparative Study.

[30] Itoh T., Kawabe M., Nagase T., Endo K., Miyoshi M., Miyahara K. (2016). Body surface area measurement in laboratory miniature pigs using a computed tomography scanner. J. Toxicol. Sci..

[31] Branski L.K., Mitterr R., Herndon D.N., Norbury W.B., Masters O.E., Hofmann M., Traber D.L., Redl H., Jeschke M.G. (2008). A porcine model of full-thickness burn, excision and skin autografting. Burns.

[32] Davis M., Baird D., Hill D., Layher H., Akin R. (2021). Management of full-thickness skin grafts. Proc. Bayl. Univ. Med. Cent..

[33] American Veterinary Medical Association (2020). AVMA Guidelines for the Euthanasia of Animals: 2020 Edition.

[34] Bergman I. (2026). Mean-Intensity-Fluorescence Macro for Immunofluorescence Images in Fiji.

[35] Alexander W. (2010). American Society of Clinical Oncology, 2010 Annual Meeting and Rose Bengal: From a Wool Dye to a Cancer Therapy. Pharm. Ther..

[36] Thompson J.F., Agarwala S.S., Smithers B.M., Ross M.I., Scoggins C.R., Coventry B.J., Neuhaus S.J., Minor D.R., Singer J.M., Wachter E.A. (2015). Phase 2 Study of Intralesional PV-10 in Refractory Metastatic Melanoma. Ann. Surg. Oncol..

[37] Kotwal G.J., Chien S. (2017). Macrophage Differentiation in Normal and Accelerated Wound Healing. Results Probl. Cell Differ..

[38] MacLeod A.S., Mansbridge J.N. (2016). The Innate Immune System in Acute and Chronic Wounds. Adv. Wound Care.

[39] Zhao E., Tang X., Li X., Zhao J., Wang S., Wei G., Yang L., Zhao M. (2025). Bioactive multifunctional hydrogels accelerate burn wound healing via M2 macrophage-polarization, antioxidant and anti-inflammatory. Mater. Today Bio.

[40] Sullivan T.P., Eaglstein W.H., Davis S.C., Mertz P. (2001). The Pig as a Model for Human Wound Healing. Wound Repair Regen..

[41] Hunt M., Torres M., Bachar-Wikstrom E., Wikstrom J.D. (2024). Cellular and molecular roles of reactive oxygen species in wound healing. Commun. Biol..

[42] Vanerio N., Stijnen M., de Mol B.A.J.M., Kock L.M. (2019). Biomedical Applications of Photo- and Sono-Activated Rose Bengal: A Review. Photobiomodul. Photomed. Laser Surg..

[43] Taraszkiewicz A., Fila G., Grinholc M., Nakonieczna J. (2013). Innovative Strategies to Overcome Biofilm Resistance. BioMed Res. Int..

[44] Merikansky S., Mercado C., Durkee H., Kobus R., Navia J.C., Arboleda A., Aguilar M.C., Martinez J.D., Flynn H.W., Miller D. (2025). Rose Bengal photodynamic antimicrobial therapy as an adjunct treatment for Pseudomonas aeruginosa infectious necrotizing scleritis. Photodiagn. Photodyn. Ther..

[45] Wang J., Li Y., Han X., Zhang H., Fan A., Yao X., Tang B., Zhang X. (2021). Light-Triggered Antibacterial Hydrogels Containing Recombinant Growth Factor for Treatment of Bacterial Infections and Improved Wound Healing. ACS Biomater. Sci. Eng..

[46] Jay J.W., Palackic A., Prasai A., Seigel Q., Siddiqui R., Bergman I., Wolf S.E., Wilkerson M.G., El Ayadi A. (2024). Photoactivated rose bengal mitigates a fibrotic phenotype and improves cutaneous wound healing in full-thickness injuries. Wound Repair Regen..

[47] Dalleau S., Baradat M., Guéraud F., Huc L. (2013). Cell death and diseases related to oxidative stress:4-hydroxynonenal (HNE) in the balance. Cell Death Differ..

[48] Ansari M., Darvishi A. (2024). A review of the current state of natural biomaterials in wound healing applications. Front. Bioeng. Biotechnol..

[49] Nasra S., Patel M., Shukla H., Bhatt M., Kumar A. (2023). Functional hydrogel-based wound dressings: A review on biocompatibility and therapeutic efficacy. Life Sci..

[50] Zonies D., Mack C., Kramer B., Rivara F., Klein M. (2010). Verified Centers, Nonverified Centers, or Other Facilities: A National Analysis of Burn Patient Treatment Location. J. Am. Coll. Surg..

